# An Extended k-Surface Framework for Electromagnetic Fields in Artificial Media

**DOI:** 10.3390/ma14247842

**Published:** 2021-12-18

**Authors:** Octavian Dănilă, Ana Bărar, Marian Vlădescu, Doina Mănăilă-Maximean

**Affiliations:** 1 Physics Department, Polytechnic University of Bucharest, 060042 Bucharest, Romania; doina.manaila@upb.ro; 2 Department of Electric Engineering and Reliability, Polytechnic University of Bucharest, 060042 Bucharest, Romania; ana.barar@upb.ro (A.B.); marian.vladescu@upb.ro (M.V.)

**Keywords:** artificial crystals, metamaterials, optical materials, electromagnetic materials

## Abstract

The complete understanding of the electromagnetic field characteristics in artificially created bulk or thin media is essential to the efficient harnessing of the multitude of linear and nonlinear effects resulting from it. Due to the fact that recently developed artificial metastructures exhibit controllable electric and magnetic properties that are completely different from natural ones, the spectrum of behavior resulting from subjecting such media to electromagnetic fields has to be revisited. In this paper, we introduce a k-surface framework that offers complete information on the dispersion properties of media with designer electric and magnetic responses with positive and negative values, as well as for the coupling between the two. The extension from the classic k-surface case resides in the consideration of magnetic and bianisotropic materials with positive and negative permittivity and permeability values, as well as the introduction of the chirality coefficient.To illustrate the applicability of our framework, we have investigated the conditions to obtain collinear second harmonic generation in the case of artificial media with positively and negatively valued electric and magnetic responses. As expected, the phase matching tuning curves, defined as the intersections between the **k**-surfaces at both frequencies, are significantly modified with respect to the classic ones.

## 1. Introduction

The control of optical effects including refraction, reflection, state of polarization description, interference, pulse creation and manipulation, energy transfer, harmonics generation and frequency mixing are a direct consequence of the interaction between the electromagnetic field and material media [1]. The characterization of the interaction relies on the knowledge of the phase of the wave, which in turn is determined by the dispersion properties of the medium that the wave travels in. This information is contained in the $k\left(\omega \right)$ function. In isotropic media, this function is independent of direction, and provides a single function, whereas in anisotropic media, the $k\left(\omega \right)$ function becomes direction-dependent, and generates an entire function set. The dispersion function also characterizes the different propagation velocities inside the media, which are both frequency- and direction-dependent in anisotropic media. Refraction at an interface occurs as a result of the configuration of the $k\left(\omega \right)$ function, in such a way as to obey the phase matching principle between the transmitted and the incident wave. The accumulated phase differences are responsible for the determination of the state of polarization, as well as delays between spectral components and natural broadening in the case of electromagnetic pulses. The dispersion function is determined by the electromagnetic response of the medium; namely, the electric permittivity ϵ and the magnetic permeability μ. In conventional optical media, the magnetic response is considered negligible, i.e., $\mu =\mu_{0}$, and only the electrical response determines the dispersion surface. This behavior leads to the classic description of the electromagnetic field, with its associated set of optical effects. However, the boundaries imposed by such materials have been extended during the last two decades, after the experimental realization of artificial, negative refractive index materials (NIM-s) [2,3,4], based on the theoretical considerations formulated almost half a century before [5]. In such media, the simultaneously obtained values for the electric permittivity ϵ and the magnetic permeability μ lead to an inversion of the conventional sign between the electric and magnetic field vectors E and H respectively, and therefore lead to an inversion of the wave vector k, while not affecting the direction of the Poynting vector S. As a cause of this inversion, the behavior of the systems in the interaction with the electromagnetic field is also flipped, resulting in a whole set of possible applications [6,7,8]. The physical realization of negative-index materials has been possible with the help of metamaterials [9] and metasurfaces [10], which are artificially-created electromagnetic field scaterrers, in which various elements and geometries are combined in order to provide a custom response. From a construction point of view, metastructures are assembled from individual elements, such as conductors or dielectrics that are deposed on a substrate. The unit cell is defined as the individual scatterer that is created by the specific combination of elements. The response of the unit cell is dependent on the geometric shape and size of the elements, their relative coordinates as well as the bulk properties of the materials that come into the composition of the elements and the substrate. The spectral response of such a unit cell differs considerably from that of the composing elements, and can provide a relatively-large amount of customization of the electromagnetic field properties (polarization spatial phase controllers [11,12], frequency-selective surfaces [13,14], giant magnetoresistance-based devices [15,16], high-resolution imaging below the diffraction limit [17,18], second harmonic generators [19,20,21]), including the exotic effects introduced by a negative refractive index (e.g., generalized reflection and refraction [22,23], object cloaking in the radio frequency and optical regimes [24,25], hyperbolic wave front generators [26,27], dispersion sign controllers [28,29,30], Huygens surfaces [31,32,33,34]). Regardless of their nature, metastructures violate the principle of locality, which states that the electric and magnetic properties of a material are the same in all directions and for all positions of that material. The locality principle also sets the general rules for obtaining normal dispersion of bulk materials in any direction. Contrarily, the nonlocality of metastructures induces a new degree of freedom in designing materials with $\epsilon \left(\omega \right)$ and $\mu \left(\omega \right)$ tailored for a certain direction. A more comprehensive discussion on the principle of locality and its relation to metasurfaces has previously been reported [35]. A first observation is that most NIMs have a non-negligible magnetic permeability, and therefore cannot be treated in the same manner as bulk optics, where the magnetic response is not taken into consideration when determining compatible propagation modes, associated wave vectors, optic axes, and states of polarization. Moreover, the presence of a non-negligible magnetic field response introduces couplings between the electric and magnetic field components, which mandates that the equations describing the electromagnetic field should be revised in the bianisotropic model [36,37,38]. Furthermore, these couplings introduce a certain chirality, which exerts itself in the fact that the scattering behavior of the metastructure changes as a function of the input polarization [39,40].

In this paper, we propose the introduction of an extended framework to describe the dispersion properties of artificial media, in which the electric, magnetic and cross-dependent responses are considered. The framework takes into account both positively and negatively valued ϵ and μ, which can easily be obtained in artificial media, such as metal-dielectric and all-dielectric metasurfaces. The cross-dependent responses are modeled by the chirality factor γ expressed by the bianisotropic model under rotational symmetry. Our approach offers a more general intuitive characterization of the electric and magnetic response through the visualization of the associated k-surfaces, and constitutes a general recipe for the design of the electric and magnetic response in such a way as to obtain the desired effect via the study of the associated k-surface. This approach can also be viewed as an extension of previous studies that treat hyperbolic materials [41] by offering information on the spatial dispersion for an extra set of configurations of permittivity and permeability, as well as chiral coefficient.For the linear optical regime, the study reveals new directions for the optical axes, as well as significant modifications to the classic **k**-surface sheets associated with biaxial and uniaxial crystals. The study of nonlinear properties focuses on determining the phase matching directions in the special case of collinear three-wave mixing, for positive and negative dispersive of artificial uniaxial media. The existence of such special properties leads to the realization of improved nonlinear optical media, which can combine phase matching with negative refractive index associated effects. Related to recent experiments, in which growth methods such as colloidal self-assembly and micelle-directed seeded growth are reported as high-yield solutions for obtaining magnetic metasurfaces [42], mechanically tunable chiral metasurfaces [43] and gold nanorods that exhibit chirality [44], our framework is especially suitable due to the fact that the properties of the metasurfaces can be determined prior to experiment, by inserting the estimated ϵ, μ and chirality factors, and evaluating the resulting **k**-surfaces. Depending on the type of dispersion that the metasurface exhibits, these evaluations can be done with multiple frequencies, in order to highlight the possibility of obtaining nonlinear effects by obtaining the appropriate phase-matching condition. The frequency regime can be shifted from the optical to the terahertz regime, where the framework can also be used in the study of newly reported broadband all-dielectric metasurfaces [45,46], subsequent devices [47] and highly sensitive metasurfaces [48]. Moreover, the obtained **k**-surface description can be useful for determining the polarization state of the propagation mode, by solving the associated Helmholtz equation in which the wave-vector is determined by all ϵ, μ and chirality factor, rather than just the positively valued ϵ as is the case in classic materials. Another possible use for the design is to help with deep-learning algorithms that enable transition from the estimate **k**-surface to the one that is specific to the architecture [49]. The net advantage of our framework is that it offers a clear, graphical solution, in the form of a modified **k**-surface, which can be easily evaluated in order to easily evaluate the optical properties of the artificial media under consideration.

## 2. Framework Description

### 2.1. Nonmagnetic Dielectrics

The classic description of the electromagnetic field is given by Maxwell’s equations, that relate both the intra- and inter-field dependencies with respect to each other:

(1) $$\begin{array}{cc} \nabla \cdot D=\rho ; & \nabla \cdot B=0; \end{array}$$

(2) $$\begin{array}{cc} \nabla \times E=-\frac{\partial B}{\partial t}; & \nabla \times H=J+\frac{\partial D}{\partial t}; \end{array}$$

(3) $$\begin{array}{cc} D=\hat{\epsilon }E; & B=\hat{\mu }H \end{array}$$

where **E** and **H** are the electric and magnetic field intensities, **D** and **B** are the electric and magnetic field inductions, ρ is the charge distribution across the volume of the region in which the equations are applied, **J** is the electric current density across the surface element of interest, $\hat{\epsilon }$ and $\hat{\mu }$ are the electric permittivity and magnetic permeability tensors, respectively, and $\nabla =\sum_{1,2,3}\left(\frac{\partial }{\partial x_{j}}e_{j}\right)$ is the first-order differential field operator. Here, we have denoted $x_{1,2,3}$ a cartesian reference frame, which can also be the *x,y,z* frame. For solid dielectrics, which are almost unanimously used in optics, the directional symmetry implies that in the relative pertmitivitty tensor we will have $\epsilon_{ij}=\epsilon_{ji}$, which reduces the number of independent variables from nine to six. Moreover, symmetric tensors are fully diagonalizable, which means that there will always exist a reference frame $\left\{x_{1},x_{2},x_{3}\right\}$, in which only the diagonal components $\epsilon_{jj}$ are nonzero. For any symmetric tensors, we have the relation [1]:

(4) $$\sum_{j=1,2,3}\epsilon_{jj}x_{j}^{2}=1$$

which is geometrically described by an ellipsoid of axes $\epsilon_{jj}$. For two equal $\epsilon_{jj}$, the surface becomes a revolution ellipsoid having the symmetry axis along the third component. Moreover, positive $\epsilon_{jj}$ represent the construction basis of the well-known refractive index ellipsoid. Another limiting property of common bulk dielectrics in the interaction with the optical field is locality, in which the properties exhibited at one point in the medium are preserved throughout the whole volume. Common dielectrics exhibit locality by preserving the same positive (or negative) dispersion function $\epsilon \left(\omega \right)$ throughout the material, and in all directions. While this property is of use in most optical application, when designing custom-response structures, locality imposes a limitation on the response.

An intuitive representation scheme of the dispersion properties is *the k-surface* which offers information on the wave vector experimented by a wave at frequency ω traveling along a certain direction in the medium. Assuming a spatial region with no charges and no currents (i.e., ρ=0 and J=0), a non-magnetic material ($\hat{\mu }=\mu_{0}$ − vacuum permeability), as well as a plane wave solution of both fields, the spatial component of Equation (2) is written as:

(5) $$\begin{array}{cc} k\times E=-\omega B; & k\times H=\omega D \end{array}$$

which transforms into:

(6) $$\begin{array}{cc} k\times E=-\omega \hat{\mu }H; & k\times H=\omega \hat{\epsilon }E \end{array}$$

when considering the material relations between each field component. After inserting the material relations (3) into the above relations, keeping in mind that for nonmagnetic media, we have $B=\mu_{0}H$ and applying the ‘k×’ operator on the first relation of the two, we obtain:

(7) $$k\times \left(k\times E\right)+\omega^{2}\mu_{0}\hat{\epsilon }E=0$$

The above equation represents a three-dimensional equation system along axes $x_{1},x_{2},x_{3}$. We take a symmetric medium, which is generally the case in natural and artificial media, we denote $k_{1},k_{2}$ and $k_{3}$ as the wave numbers along these directions and we introduce the pseudotensor associated with the vector product. For a vector A, the vector product writes as:

(8) $$k\times A=\left[\begin{array}{ccc} 0 & k_{3} & -k_{2}\\ -k_{3} & 0 & k_{1}\\ k_{2} & -k_{1} & 0 \end{array}\right]\cdot \left[\begin{array}{c} A_{x}\\ A_{y}\\ A_{z} \end{array}\right]=\hat{K}A$$

With the above considerations, the matrix form of Equation (7) is:

(9) $$\left[\begin{array}{ccc} \mu_{0}\omega^{2}\epsilon_{11}-k_{2}^{2}-k_{3}^{2} & k_{1}k_{2} & k_{1}k_{3}\\ k_{1}k_{2} & \mu_{0}\omega^{2}\epsilon_{22}-k_{1}^{2}-k_{3}^{2} & k_{2}k_{3}\\ k_{1}k_{3} & k_{2}k_{3} & \mu_{0}\omega^{2}\epsilon_{33}-k_{1}^{2}-k_{2}^{2} \end{array}\right]\left[\begin{array}{c} E_{1}\\ E_{2}\\ E_{3} \end{array}\right]=0$$

We can also introduce a compacted form for the above relation, by introducing the operator ${\hat{\Gamma }}_{0}={\hat{K}}^{2}+\omega \mu_{0}\hat{\epsilon }$ as the non-magnetic, non-chiral operator. Combining Equations (7) and (8), we obtain:

(10) $$\left({\hat{K}}^{2}+\omega \mu_{0}\hat{\epsilon }\right)E=0$$

**Figure 1:**
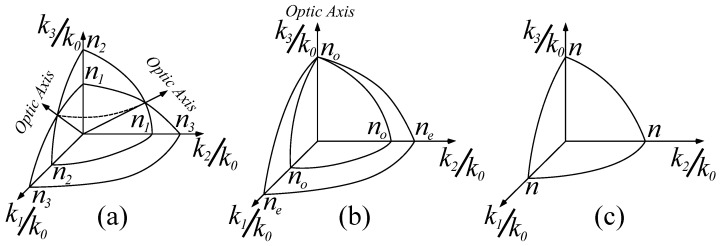
Schematic illustration of the **k**-surfaces for the classic case of nonmagnetic dielectrics: (**a**) a biaxial crystal with refractive indices $n_{1}$, $n_{2}$ and $n_{3}$, in which the optic axis is the intersection of the two **k**-surface sheets; (**b**) a uniaxial crystal with refractive indices $n_{o}$ and $n_{e}$ in which the intersection between the two sheets is a single point per hemisphere which is parallel to *Oz*; (**c**) an isotropic medium with a refractive index *n*. The two sheets are overlapped on a single spherical surface, and any direction constitutes an optic axis.

In order to obtain non-trivial solutions for the electric field, the determinant of the matrix in the above equation has to equal zero. We define the vacuum wave number as $k_{0}=\omega \sqrt{\epsilon_{0}\mu_{0}}$, and we normalize the values defining the k-surface to it. For positive values of $\epsilon_{jj}$, this condition is satisfied by a two-sheet surface in the k-space defined by axes $k_{1},k_{2}$ and $k_{3}$. The direction $u=\left(u_{1},u_{2},u_{3}\right)$ defined by the origin and any point on the k-surface yields the wave vector $k=\frac{\omega }{c}\sqrt{\epsilon }$, where ϵ is the permittivity perceived by the electric field as the wave propagates parallel to it. The intersection between the two sheets defines the optic axis, which represents the privileged direction in which the wave attains the same velocity regardless of polarization. When $\epsilon_{11}\neq \epsilon_{22}\neq \epsilon_{33}$, the optic axis is contained in the $Ox_{1}x_{3}$ plane, and the configuration defines a biaxial crystal. For $\epsilon_{11}=\epsilon_{22}\neq \epsilon_{33}$, the optic axis is parallel to the $x_{3}$ axis, defining a uniaxial crystal. When all three components are equal, the medium is isotropic, and any direction in space can be considered an optic axis. The property of the optic axis is that any wave that propagates parallel to it will have its associated polarization components experiencing the same permittivity, and implicitly the same refractive index in the case of natural dielectrics. For any other direction, the permittivity values that are experienced by the field modes are different, and lead to a phase difference and an overall modification of the state of polarization. A schematic illustration is presented in Figure 1.

### 2.2. Magnetic Dielectrics

While conventional optical media exhibit little to no magnetic activity, artificially designed media such as metasurfaces can be engineered to exhibit a non-negligible, designer magnetic permeability tensor. This brings significant modifications to the solutions of Maxwell’s Equations (2) and (3), which for harmonic solutions, now write as:

(11) $$\begin{array}{cc} \hat{K}E=-\omega \hat{\mu }H; & \hat{K}H=\omega \hat{\epsilon }E \end{array}$$

we multiply to the left by the inverse permeability tensor ${\hat{\mu }}^{-1}$ in the first equation of set (11), and we obtain:

(12) $$H=-\frac{1}{\omega }{\hat{\mu }}^{-1}\hat{K}E$$

We introduce into the second relation of set (11), and we obtain:

(13) $$\left(\hat{K}{\hat{\mu }}^{-1}\hat{K}+\omega^{2}\hat{\epsilon }\right)E=0$$

Just as before, we can write the above equation in a compacted form by introducing the magnetic permeability-dependent operator ${\hat{\Gamma }}_{\mu }=\hat{K}{\hat{\mu }}^{-1}\hat{K}+\omega^{2}\hat{\epsilon }$. To produce nontrivial solutions, the determinant of ${\hat{\Gamma }}_{\mu }$ must be equal to zero, an equation that can be satisfied by a certain k-surface in the $\left(k_{1},k_{2},k_{3}\right)$ basis. The other considerations such as the determination of the optic axis, associated field polarizations and energy flow direction remain unchanged.

### 2.3. Bianisotropic Media

In the most general case, materials that are responsive to both electric and magnetic fields also possess an extra set of coefficients, that account for the modifications induced by the electric field to the magnetic field, and vice versa. Experimentally, due to the fact that typical crystals are non-magnetic, the couplings between the electric and magnetic field that compose the wave have been introduced externally, at a much lower frequency, in order to actively rotate the polarization plane of the electric field. The influence of the external electric field on the wave polarization (Kerr and Pockels effects) is by default included in the rewriting of the electric response ϵ, but an external magnetic field influence of the polarization plane has to be accounted by a coupling coefficient of the material, that relates the two fields. For Faraday rotators, which are devices that operate based on this effect, the coupling coefficient is known as the Verdet constant. In a general framework, the couplings between the electric and magnetic fields by means of material responses are supplied intrinsically by means of material design, and therefore can realize the desired influences directly. In our framework, these couplings are modeled by introducing two tensors $\hat{\alpha }$ and $\hat{\beta }$, which account for the electric-to-magnetic field and magnetic-to-electric field influences, respectively. The introduction of these tensors in the Maxwell equations picture is known as the bianisotropic model of the electromagnetic field. Due to the fact that both fields are harmonic and even for artificial media, the designer symmetries impose an identical response of the field couplings; it is safe to assume that $\hat{\alpha }=\hat{\beta }$, but for the sake of completeness, we will consider them independently in the framework. The Maxwell equations framework for harmonic solutions viewed in this new picture reads as:

(14) $$\begin{array}{cc} \hat{K}E=-\omega \left(\hat{\mu }H+\hat{\beta }E\right); & \hat{K}H=\omega \left(\hat{\epsilon }E+\hat{\alpha }H\right) \end{array}$$

In the first equation of set (14), we multiply to the left with ${\hat{\mu }}^{-1}$ and obtain:

(15) $${\hat{\mu }}^{-1}\hat{K}E=-\omega \left({\hat{\mu }}^{-1}\hat{\beta }E+H\right)$$

which leads to:

(16) $$H=-\frac{1}{\omega }{\hat{\mu }}^{-1}\hat{K}E-{\hat{\mu }}^{-1}\hat{\beta }E$$

We substitute this form into the second equation of the set (14), and obtain:

(17) $$-\frac{1}{\omega }\hat{K}{\hat{\mu }}^{-1}\hat{K}E-\hat{K}{\hat{\mu }}^{-1}\hat{\beta }E=\omega \hat{\epsilon }E-\hat{\alpha }{\hat{\mu }}^{-1}\hat{K}E-\omega \hat{\alpha }{\hat{\mu }}^{-1}\hat{\beta }E$$

Since the electric field component is the only remaining vector to be operated on, we can omit it as we convert the above relation to an operator form. Moreover, we forcibly multiply by ω and obtain:

(18) $${\hat{\Gamma }}_{b}=\omega^{2}\left(\hat{\alpha }{\hat{\mu }}^{-1}\hat{\beta }-\hat{\epsilon }\right)+\omega \left(\hat{\alpha }{\hat{\mu }}^{-1}\hat{K}-\hat{K}{\hat{\mu }}^{-1}\hat{\beta }\right)-\hat{K}{\hat{\mu }}^{-1}\hat{K}$$

with the associated operator equation ${\hat{\Gamma }}_{b}E=0$. Again, to produce nontrivial solutions, the determinant of the operator has to be zero. This condition produces the associated k-surfaces that establishes the spatial properties of the wave during propagation.

### 2.4. Phase Matching

The propagation behavior of electromagnetic fields in media is directly derived from both wave and medium properties. Following Fermat’s principle of least time, whenever the electromagnetic properties of the medium are changed at an interface, the phase of the transmitted wave has to match the phase of the incident wave. This condition is known as ‘phase matching’, and it constitutes the basis of any propagation behavior at interfaces between media. A general discussion of phase matching and its effects in general reflection and refraction in material media can be found in references [1,22]. In the case of an interface between isotropic media with ($\epsilon_{1}$, $\mu_{1}$) and ($\epsilon_{2}$, $\mu_{2}$), the wave numbers of the incident and transmitted waves write as:

(19) $$\begin{array}{cc} k_{1}=\frac{\omega }{v_{1}}=\frac{\omega }{c}\sqrt{\epsilon_{1}\mu_{1}} & k_{2}=\frac{\omega }{v_{2}}=\frac{\omega }{c}\sqrt{\epsilon_{2}\mu_{2}} \end{array}$$

The directions of the wave vectors that have the above sizes are configured as a result of the phase matching condition: to ensure zero phase difference between the incident and transmitted waves, the wave vector components that are parallel to the interface have to be equal. This leads to Snell’s law of refraction:

(20) $$k_{1}\sin\theta_{1}=k_{2}\sin\theta_{2}$$

where $\theta_{1}$ and $\theta_{2}$ are the incident and transmission angles, respectively. The same considerations can be applied for anisotropic media, where the direction of the incident wave imposes the specific values of medium response (ϵ, μ) that are experienced by it. Nonlinear phenomena such as three-wave mixing rely on the combination of two waves at frequencies $\omega_{a}$ and $\omega_{b}$ which interact in a certain region of a nonlinear medium that exhibits second-order crystalline asymmetry (second-order nonlinear medium), resulting in the generation of a third wave at $\omega_{c}$. The process is time-reversed, meaning that there is also the possibility of obtaining waves $\omega_{a}$ and $\omega_{b}$ from propagating wave $\omega_{c}$ inside the second-order nonlinear medium. Regardless of the desired nonlinear mixing, assuming $\omega_{a}>\omega_{b}$, the process must obey the phase matching condition, which in this case transforms into the following:

(21) $$\begin{array}{cc} \omega_{c}=\omega_{a}\pm \omega_{b}; & k_{c}=k_{a}+k_{b} \end{array}$$

Of the two above relations, the first represents the energy conservation condition, and the second one represents the phase matching condition. The second relation generates the direction and the spatial frequencies that are allowed to be created by the nonlinear medium. The configuration of the directions and spatial frequencies has to also take into account the energy conservation relation, which adds another constraint to the problem. As an example, the second harmonic generation process along a single direction (collinear SHG) can be viewed as two waves having the same fundamental frequency $\omega_{a}=\omega_{b}=\omega$ that interact in a second-order nonlinear medium to produce an up-converted signal $\omega_{c}=2\omega$. When considering an uniaxial nonmagnetic medium (i.e., $\epsilon_{11}=\epsilon_{22}=\epsilon \neq \epsilon_{33}$ and $\hat{\mu }=\hat{I}$, where $\hat{I}$ is the identity matrix), the phase matching condition becomes:

(22) $$\sqrt{\epsilon \left(\omega \right)}=\sqrt{\epsilon \left(2\omega \right)}$$

For an isotropic medium, this condition is impossible to realize due to the naturally dispersive behavior enforcing that $\epsilon \left(2\omega \right)>\epsilon \left(\omega \right)$. However, for uniaxial crystals, the mixing can still be performed by appropriately choosing the ordinary and extraordinary directions of propagation in such a way as to satisfy Equation (22).

At any given frequency, bulk materials exhibit either positive or negative dispersion for both electric and magnetic properties. However, due to the fact that artificial media can be tailored in order to obtain designer dispersion properties for each property, the phase matching condition for nonlinear processes can be significantly altered by means of independent conditions on the dispersion properties. Therefore, the introduction of artificial media with controllable properties relaxes the phase matching condition by introducing four degrees of freedom, in the form of sign control of ϵ and μ, as well as control of the dispersion properties of the two properties. For example, in such an artificial media, the collinear SHG condition given by Equation (22) is changed to:

(23) $$\sqrt{\epsilon \left(\omega \right)\mu \left(\omega \right)}=\sqrt{\epsilon \left(2\omega \right)\mu \left(2\omega \right)}$$

and while the dispersion behavior of each electromagnetic field property ensures that for isotropic media there can be no situation in which $\epsilon \left(\omega \right)=\epsilon \left(2\omega \right)$ or $\mu \left(\omega \right)=\mu \left(2\omega \right)$, Equation (22) can be satisfied if one of the parameters exhibits a controllable dispersion behavior (either positive or negative) in such a way as to compensate the dispersion behavior of the other parameter. This approach can be extended for nonlinear three-wave mixing configurations, in which the directions of the waves are configured based on the compatibility with the medium properties and the phase matching condition. For bianisotropic media, the introduction of the chirality tensor further complicates the phase matching condition, and the wave vector matching condition is solvable by numeric and graphic methods. Lastly, the non-local characteristic of artificially-created media imply that the dispersion properties are not obeyed in the entire volume or even in points along the same axis. This leads to a case-by-case study of the dispersion properties, where the direction of the incident wave as well as the coordinates of incidence on the material can strongly influence the dispersion characteristics.

## 3. Results and Discussion

The obtained **k**-surfaces are the nontrivial solutions of the equation set $\hat{\Gamma }E=0$ at a single angular frequency ω, where the operators $\hat{\Gamma }$ can be either ${\hat{\Gamma }}_{0}$, ${\hat{\Gamma }}_{\mu }$ or ${\hat{\Gamma }}_{b}$. Although it is fundamentally established, we have also reviewed the classic model, in which all three permittivity coefficients are positive for establishing completeness of our framework as well as to validate the results obtained in our calculations.

### 3.1. Nonmagnetic Dielectrics

**Figure 2:**
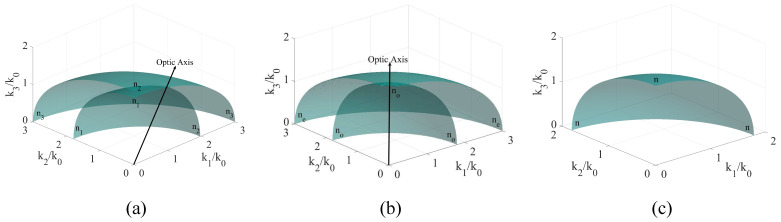
Classic **k**-surfaces for: (**a**): A biaxial crystal with $\epsilon_{11}=3.22$, $\epsilon_{22}=3.84$ and $\epsilon_{33}=9$, for which the two surfaces intersect in one point in the $Ox_{1}x_{3}$ plane. When interchanging the values of $\epsilon_{11}$ and $\epsilon_{22}$, the **k**-surface does not change its shape, rather the axes $k_{1}$ and $k_{2}$ are interchanged; (**b**): A uniaxial crystal with $\epsilon_{11}=\epsilon_{22}=3.22$ and $\epsilon_{33}=9$ for which the two surfaces intersect in one point along the $x_{3}$ axis; (**c**): An isotropic crystal with $\epsilon_{11}=\epsilon_{22}=\epsilon_{33}=3.22$ for which the two surfaces are identical for all points in space. For all cases, the values of the permittivity components are arbitrary, which reinforces the general validity of the model. The optic axis is the direction defined by the origin of the coordinate system and the intersection of the two surfaces.

For non-magnetic, non-chiral materials, the dispersion properties are obtained by solving the equation ${\hat{\Gamma }}_{0}E=0$. When $\epsilon_{11}\neq \epsilon_{22}$, a biaxial medium is obtained, with an optical axis in the $k_{1}k_{3}$ plane for $\epsilon_{11}<\epsilon_{22}$ and in the $k_{2}k_{3}$ plane for $\epsilon_{11}>\epsilon_{22}$. When $\epsilon_{11}=\epsilon_{22}$, the optical axis becomes parallel to the $k_{3}$ axis, describing a uniaxial medium. When all three permittivities are equal, the medium is isotropic, and any direction in space can serve as an optical axis. The k-surfaces describing these cases are presented in Figure 2, in which, for the purpose of maintaining generality of our method, the values of ϵ have been conveniently chosen for showcasing the effect, rather than being assigned the values of specific materials.

In terms of field properties, the sheets forming the **k**-surface offer information on the velocity and phase attained by any polarization mode. For biaxial media, the **k**-surface is composed of two sheets, that form two ellipsoids of revolution centered around the origin. The propagation direction serves as the director of an oriented plane, whose intersection with the two sheets gives the values of the refractive indices experienced by any polarization mode. In the case of uniaxial media with positive permittivity values, one sheet of the **k**-surface becomes an ellipsoid of revolution, while the other one becomes a sphere. The sphere describes the ordinary, while the ellipsoid describes the extra-ordinary propagation mode. In the case of isotropic media, the two sheets are overlapped, resulting in a single mode of propagation in which the refractive index experienced by all polarizations is the same regardless of the propagation direction.

**Figure 3:**
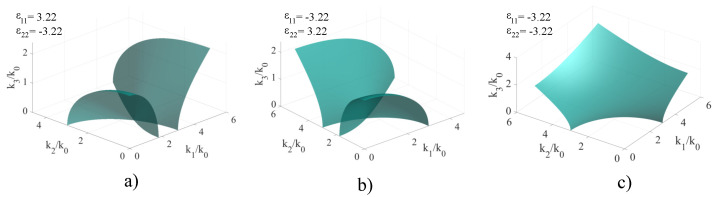
Extended **k**-surface for: (**a**) A nonmagnetic, non-chiral uniaxial crystal with $\epsilon_{11}=3.22$, $\epsilon_{22}=-3.22$ and $\epsilon_{33}=9$; (**b**) A nonmagnetic, non-chiral uniaxial crystal with $\epsilon_{11}=-3.22$, $\epsilon_{22}=3.22$ and $\epsilon_{33}=9$; (**c**) A nonmagnetic, non-chiral uniaxial crystal with $\epsilon_{11}=\epsilon_{22}=-3.22$ and $\epsilon_{33}=9$.

In the case of artificial media, the signs of $\epsilon_{11}$ and $\epsilon_{22}$ can be tailored to be either positive or negative. In the case of simultaneously negative $\epsilon_{11}$ and $\epsilon_{22}$, we obtain the so-called electric plasmas, which represent metals at optical frequencies [24]. The case in which just one of the permittivity coefficients has its sign reversed does not occur naturally, but can be obtained by using appropriate metasurface architectures. The conducted study assumes that $\left|\epsilon_{11}\right|=\left|\epsilon_{22}\right|$, which for positive-valued permittivity values would describe a uniaxial crystal. This special case was chosen for two reasons: firstly, uniaxial crystals are extensively used in optical applications, which enables an application-oriented comparative study, and secondly, in a biaxial crystal, the shape of the resulting **k**-surfaces would not change, but would only suffer deformations along the axis, causing asymmetry. The resulting **k**-surfaces for nonmagnetic, non-chiral artificial crystals in which the signs of $\epsilon_{11}$ and $\epsilon_{22}$ are either alternatively or simultaneously shifted between negative and positive values. The cases are represented in Figure 3.

For $\epsilon_{11}>0$ and $\epsilon_{22}<0$, the **k**-surface obtained still comprises of two sheets, but their point of intersection now lies in the $k_{1}k_{2}$ plane. The sheets are symmetric with respect to this point, and suffer a certain deformation. The symmetry plane of the surface is perpendicular to the $k_{1}k_{2}$ and $k_{1}k_{3}$ planes, and contains the point of intersection between the two sheets. This case is presented in Figure 3a. When switching the signs between $\epsilon_{11}$ and $\epsilon_{22}$, the surface is ’rotated’ with π/2 around the axis parallel with $k_{1}k_{3}$ which contains the point of intersection, with the rest of the properties remaining unchanged. This behavior is consistent with the symmetric tensor theory. This case is presented in Figure 3b. When both $\epsilon_{11}$ and $\epsilon_{22}$ are negative, we obtain a negative-index material which is characterized by a single **k**-surface describing a rotational hyperboloid instead of an ellipsoid. Moreover, there is only one sheet composing the surface instead of two, and depending on the values of the two permittivity coefficients, the ellipsoid may possess a gap region, in which there are no solutions for the wave vector. This case is presented in Figure 3c.

### 3.2. Magnetic, Non-Chiral Dielectrics

**Figure 4:**
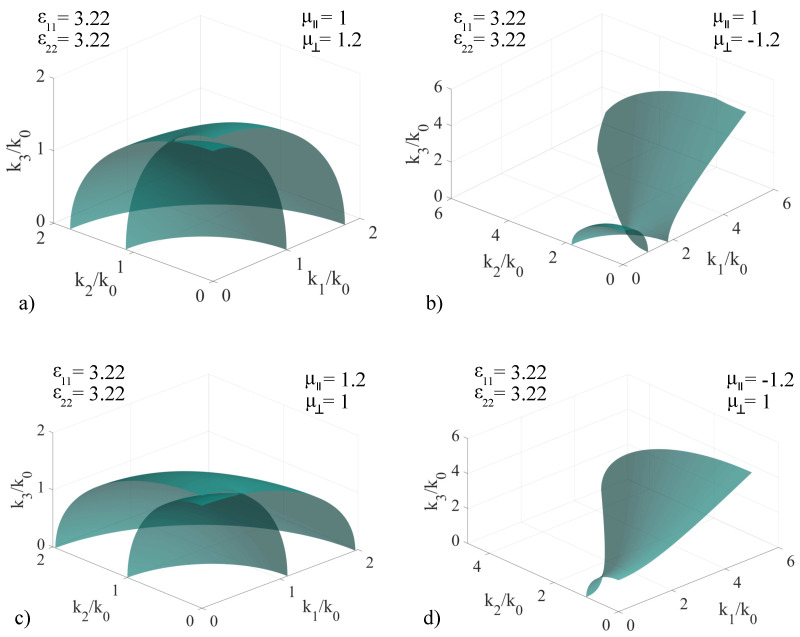
Extended **k**−surface for various configurations of non-chiral artificial media: $\epsilon_{11}=\epsilon_{22}=3.22$, $\epsilon_{33}=9$, $\mu_{⊥}$ and $\mu_{\|}=\left\{1,\pm 1.2\right\}$ cycled independently, with specific values given in the insets of the figures: (**a**) $\mu_{\|}=1$, $\mu_{⊥}=1.2$; (**b**) $\mu_{\|}=1$, $\mu_{⊥}=-1.2$; (**c**) $\mu_{\|}=1.2$, $\mu_{⊥}=1$; (**d**) $\mu_{\|}=-1.2$, $\mu_{⊥}=1$.

For determining the associated **k**-surfaces in this case, we have considered an electrically-uniaxial medium with $\epsilon_{11}=\pm 3.22$ and $\epsilon_{22}=\pm 3.22$ independently. To model the magnetic response, we have also imposed fixed values $\mu_{\|}=1,\pm 1.2$ and $\mu_{⊥}=1,\pm 1.2$, independently. The chirality coefficient is zero for all subcases considered. Just as before, to preserve generality of our method, the values considered for the response components are conveniently chosen, in order to showcase the modifications to the associated **k**-surfaces. The first study conducted assumes positive permittivity values while cycling through the fixed permeability values, and the results are presented in Figure 4, with the specific values of each sub-case indicated in the insets.

Based on the obtained results, the non-chiral version of the artificial media considered exhibit properties that differ significantly from the classic, non-magnetic positive-valued ϵ coefficients: In the ($\mu_{⊥}=1.2,\mu_{\|}=1$) case, presented in Figure 4a, we note that the magnetic property modifies the solution such that we no longer obtain a uniaxial crystal even though $\epsilon_{11}=\epsilon_{22}$. The change in the value of $\mu_{⊥}$ induces a deformation of the initially spherical sheet to an ellipsoid along the $k_{2}$ axis, as well as an inclination of the optic axis, from being parallel to the $k_{3}$ axis to a direction contained in the $k_{2}k_{3}$ plane, at a certain angle α. This change of direction is due to the fact that the intersection point of the sheets moves to a coordinate located in the $k_{2}k_{3}$ plane. When moving to the ($\mu_{\|}=1,\mu_{⊥}=-1.2$) case, pictured in Figure 4b, we obtain a surface that comprises of two sheets, one being a deformed ellipsoid and the other a deformed hyperboloid, both intersecting at a point. The surface is similar to the one presented in Figure 3b, with the difference that the coordinates of the intersection point between the two sheets shifts from (2.2, 1.9, 0) to approximately (1.8, 1.9, 0) in the ($k_{1},k_{2},k_{3}$) set, with a shift to a smaller $k_{1}$ value. For the ($\mu_{\|}=1.2,\mu_{⊥}=1$) case, pictured in Figure 4c, the **k**-surface is similar to that of the classic biaxial media, with the difference that the spherical surface is deformed to form a hyperboloid along the $k_{1}$ axis. The intersection of the two sheets is located in the $k_{1}k_{3}$ plane, and therefore the direction of the optic axis is contained in the same plane, with the same angle of inclination α with respect to the $k_{3}$ axis which characterizes the optic axis of the reference uniaxial media. In the ($\mu_{\|}=-1.2,\mu_{⊥}=1$) case, pictured in Figure 4d, the **k**-surface transforms into a single-sheet hyperboloid, which under the given parameter values covers the entire positive octant. This behavior implies that there are no forbidden parameter values in the calculation of the wave vectors.

**Figure 5:**
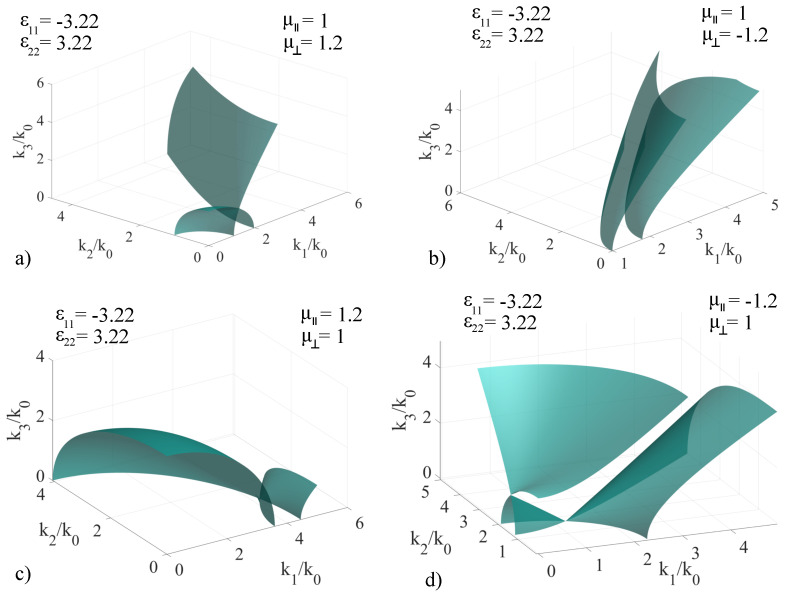
Extended **k**−surface for various configurations of non-chiral artificial media in which $\epsilon_{11}=-3.22$, $\epsilon_{22}=3.22$, $\epsilon_{33}=9$, $\mu_{⊥}$ and $\mu_{\|}=\left\{1,\pm 1.2\right\}$ are cycled independently, with specific values given in the insets of the figures: (**a**) $\mu_{\|}=1$, $\mu_{⊥}=1.2$; (**b**) $\mu_{\|}=1$, $\mu_{⊥}=-1.2$; (**c**) $\mu_{\|}=1.2$, $\mu_{⊥}=1$; (**d**) $\mu_{\|}=-1.2$, $\mu_{⊥}=1$.

The second set of calculations were performed by setting $\epsilon_{11}=-3.22$, while $\epsilon_{22}$ remains unchanged and the values of the permeability coefficients were selectively cycled through the same set of fixed values. The results are presented in Figure 5.

In the ($\mu_{\|}=1,\mu_{⊥}=1.2$) case, depicted in Figure 5a, we obtained a two-sheet surface with an intersection between them in the $k_{1}k_{3}$ plane. The two surfaces are mirrored with respect each other by means of the axis parallel to $k_{3}$ that contains the intersection point. For the ($\mu_{\|}=1,\mu_{⊥}=-1.2$), presented in Figure 5b the **k**-surface contains two sheets that do not intersect in any point. Also, the two sheets intersect the $k_{1}$ axis in $k_{1}=1$ and $k_{1}≃1.8$, which in combination with the hyperbolic shape of both surfaces allows the existence of forbidden propagation directions, where no solutions for *k* are obtained. In the ($\mu_{\|}=1.2,\mu_{⊥}=1$) case, shown in Figure 5c, the two-sheet surface is similar to the one in Figure 5a, but the intersection point of the sheets lies in the $k_{1}k_{2}$ plane and the mirroring axis is parallel to $k_{2}$ and contains the intersection point. For the ($\mu_{\|}=-1.2,\mu_{⊥}=1$) case, shown in Figure 5d, the **k**-surface contains three sheets, exhibiting two intersection points, one in the $k_{1}k_{2}$ plane and one in the $k_{1}k_{3}$ plane. The three sheets form in a manner that allows the existence of forbidden propagation directions.

**Figure 6:**
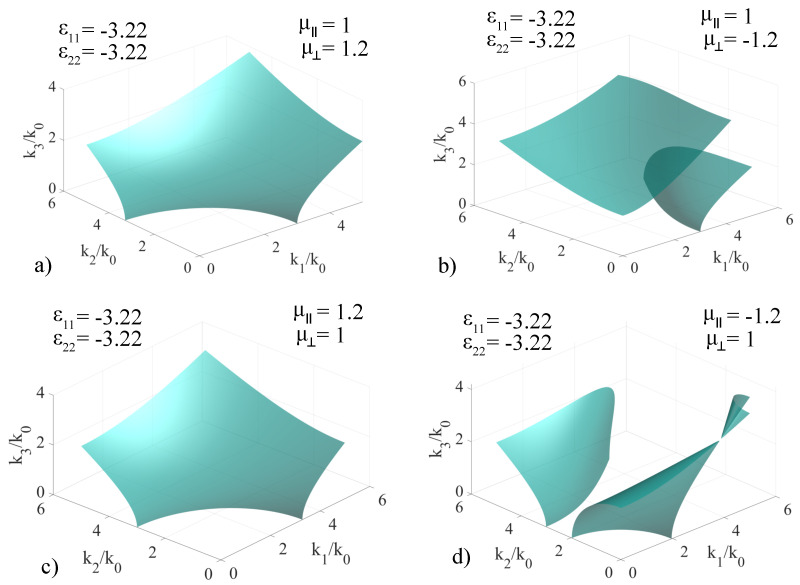
Extended **k**−surface for various configurations of non-chiral artificial media in which $\epsilon_{11}=\epsilon_{22}=-3.22$, $\epsilon_{33}=9$, $\mu_{⊥}$ and $\mu_{\|}=\left\{1,\pm 1.2\right\}$ are cycled independently, with specific values given in the insets of the figures: (**a**) $\mu_{\|}=1$, $\mu_{⊥}=1.2$; (**b**) $\mu_{\|}=1$, $\mu_{⊥}=-1.2$; (**c**) $\mu_{\|}=1.2$, $\mu_{⊥}=1$; (**d**) $\mu_{\|}=-1.2$, $\mu_{⊥}=1$.

The third set of calculations were performed by setting $\epsilon_{11}=\epsilon_{22}=-3.22$, and the values of the permeability coefficients were selectively cycled through the same set of fixed values. The results are presented in Figure 6.

For the ($\mu_{\|}=1,\mu_{⊥}=1.2$) case, depicted in Figure 6a, the associated **k**-surface consists of a single-sheet hyperboloid, which is conventionally obtained in non-magnetic negative-index media. At infinity, this sheet is asymptotic to a conical lateral surface, which implies that all the directions contained within the volume of the conical surface do not support propagation modes. For the ($\mu_{\|}=1,\mu_{⊥}=-1.2$), depicted in Figure 6b, the **k**-surface consists of two sheets which do not intersect each other, allowing for forbidden propagation modes. Furthermore, there is no direction intersecting both surfaces; therefore, regardless of direction, there is at most only one compatible propagation mode. For the ($\mu_{\|}=1.2,\mu_{⊥}=\;1$) case, depicted in Figure 6c, a hyperbolic one-sheet surface is obtained, with the same properties as the one in Figure 6a. In the ($\mu_{\|}=-1.2,\mu_{⊥}=1$) case, depicted in Figure 6d, the associated **k**-surface consists of three sheets, two of which have only one intersection point in the $k_{1}k_{3}$ plane, and the third being separated from the other two. Contrary to the case depicted in Figure 6b, however, any direction will intersect at least one of the three sheets, and therefore, there are no forbidden propagation modes. Some directions intersect only one sheet of the **k**-surface, resulting in only one compatible propagation mode.

### 3.3. Chiral, Magnetic Media

When introducing the bianisotropic components in the equations, the **k**-surfaces suffer a certain deformation from the shapes attained by their non-chiral counterparts. The deformations induced are strongly dependent on the values of the bianisotropic coefficients $\hat{\alpha }$ and $\hat{\beta }$. In the calculations, we assumed that the reciprocal influences were equal, due to the symmetry of the medium, and equal to the chirality tensor defined as:

(24) $$\hat{n}=\left[\begin{array}{ccc} 0 & 0 & 0\\ 0 & 0 & -\gamma \\ 0 & \gamma & 0 \end{array}\right]$$

where γ is the chirality coefficent. Based on this condition, the bianisotropic operator given by Equation (18) becomes:

(25) $${\hat{\Gamma }}_{b}=\omega^{2}\left(\hat{n}{\hat{\mu }}^{-1}\hat{n}-\hat{\epsilon }\right)+\omega \left(\hat{n}{\hat{\mu }}^{-1}\hat{K}-\hat{K}{\hat{\mu }}^{-1}\hat{n}\right)-\hat{K}{\hat{\mu }}^{-1}\hat{K}$$

**Figure 7:**
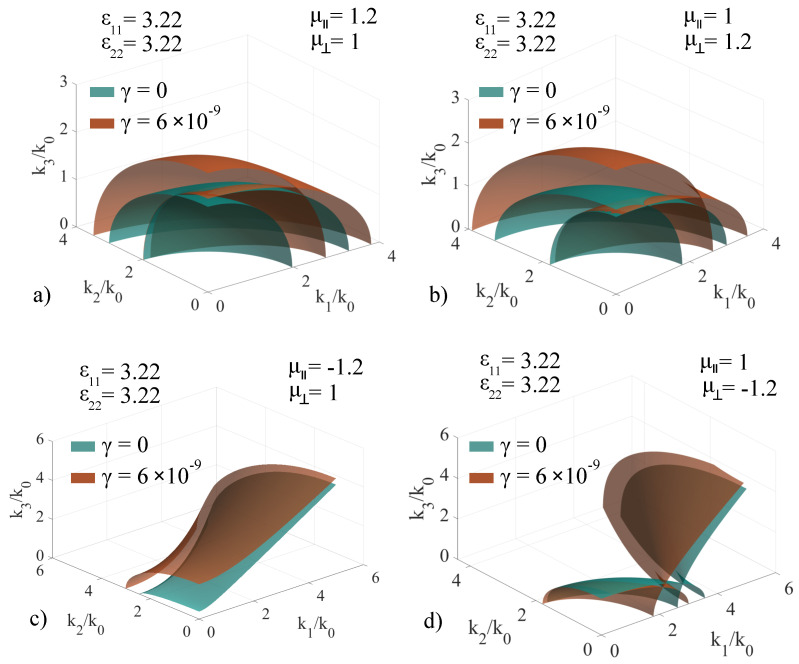
Extended **k**−surfaces for chiral magnetic media under various permittivity configurations referenced to the same non-chiral configuration, with the values of the electric and magnetic response components being specified in the insets: $\epsilon_{11}=\epsilon_{22}=3.22$, $\epsilon_{33}=9$ and: (**a**) $\mu_{\|}=1.2$, $\mu_{⊥}=1$; (**b**) $\mu_{\|}=1$, $\mu_{⊥}=1.2$; (**c**) $\mu_{\|}=-1.2$, $\mu_{⊥}=1$, (**d**) $\mu_{\|}=1$, $\mu_{⊥}=-1.2$.

The nontrivial propagation mode solutions are obtained by imposing the determinant of the operator to equal zero. Due to the fact that the chirality coefficient appears only in the case where the behavior of the magnetic field component is no longer negligible, nonmagnetic media automatically have no chirality coefficient. For magnetic media, the results are presented in a comparative manner in Figure 7, by overlapping the **k**-surfaces of the same medium in a chiral and non-chiral configuration.

Initially, we have considered $\epsilon_{11}=\epsilon_{22}=3.22$, and we have cycled $\mu_{\|}$ and $\mu_{⊥}$ between the values ±1.2 for a chirality coefficient $\gamma =6\times 10^{-9}$. The value of the chirality factor was selected in such a way as to represent clear deformations of the **k**-surfaces. In the ($\mu_{\|}=1.2$, $\mu_{⊥}=1$) case, presented in Figure 7a, the two-sheet **k**-surface is modified in such a way that both sheets are ellipsoids, and the chirality coefficient changes the direction of the optic axis from the $k_{2}k_{3}$ plane to the $k_{1}k_{3}$ plane. The chirality coefficient also introduces significant growth in the ellipsoid semiaxes values. In the ($\mu_{\|}=1$, $\mu_{⊥}=1.2$) case, depicted in Figure 7b, the two-sheet **k**-surface yields an optic axis in the $k_{1}k_{3}$ plane for both chiral and non-chiral configurations, as well as the associated modifications to the ellipsoid semiaxes. This implies that the introduction of the chirality coefficient offers reduced sensitivity to the changes in the permeability coefficients, keeping the optical axis in the $k_{1}k_{3}$ plane, regardless of the switch between the values of the permeability components. For the ($\mu_{\|}=-1.2$, $\mu_{⊥}=1$) case, presented in Figure 7c, the one-sheet hyperboloid is scaled and deformed at lower k-values. For the ($\mu_{\|}=1$, $\mu_{⊥}=-1.2$) case, presented in Figure 7d), the two-sheet surface maintains the intersection point in the $k_{1}k_{0}$ plane, but the chirality coefficient shifts it towards a lower $k_{1}/k_{0}$ coordinate. The induced deformations in this case are negligible.

### 3.4. Phase Matching

**Figure 8:**
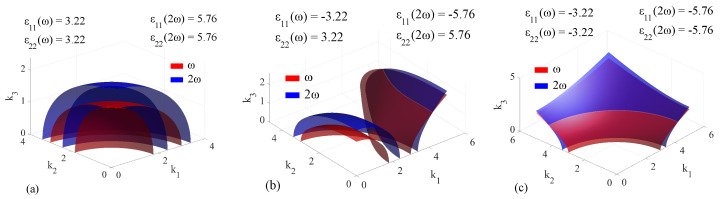
Extended **k**−surfaces for nonmagnetic artificial media in which the permittivity values are cycled between fixed values in such a way as to highlight positive dispersion. In all cases, $\epsilon_{33}\left(\omega \right)=9$ and $\epsilon_{2\omega }=10.5$. The directions corresponding to collinear phase matching are given by the intersections between a sheet at ω (red) and one at 2ω (blue). The cases are: (**a**) $\epsilon_{11}\left(\omega \right)=\epsilon_{22}\left(\omega \right)=3.22$, $\epsilon_{11}\left(2\omega \right)=\epsilon_{22}\left(2\omega \right)=5.76$; (**b**) $\epsilon_{11}=-\epsilon_{22}\left(\omega \right)=-3.22$, $\epsilon_{11}\left(2\omega \right)=-\epsilon \left(2\omega \right)=-5.76$; (**c**) $\epsilon_{11}\left(\omega \right)=\epsilon_{22}\left(\omega \right)=-3.22$, $\epsilon_{11}\left(2\omega \right)=\epsilon_{22}\left(2\omega \right)=-5.76$.

Regarding phase matching, we limited our study to the collinear generation of the second harmonic (SHG) in the same uniaxial media, in which the dispersion properties of the electric and magnetic components were independently switched from positive to negative as a function of the direction. The condition of obtaining collinear fundamental and second harmonic signals is by default incompatible with chiral media, in which the chirality tensor does not permit Equation (23) to be valid. While not discussed here, non-collinear configurations permit the use of chiral media for obtaining second harmonics in desired directions. The directions allowing collinear SHG are defined on one end by the origin of the $k_{1}k_{2}k_{3}$ representation set and on the other end by the geometric locus defined by the intersection of the sheet at the fundamental frequency ω and at the harmonic 2ω. For the case in which no such intersection exists, there is no possibility of directly achieving collinear SHG. The results for a nonmagnetic medium in which the permittivity values are set in order to highlight both positive and negative dispersion are presented in Figure 8.

Based on the obtained results, we can formulate the following discussion: In the classical, positive-valued ϵ and μ materials, the intersection between two sheets at ω and 2ω represents a circle between either the ellipsoid sheet of the ω-surface and the spherical sheet of the 2ω-surface, or the intersection between the spherical sheet of the ω-surface and the ellipsoid sheet of the 2ω-surface, depending on the positivity of dispersion. The first case presented in Figure 8a, in which $\epsilon \left(2\omega \right)>\epsilon \left(\omega \right)$ corresponds to a positive uniaxial crystal, that allows the so-called ‘ee-o’ mixing, in which two waves at frequency ω propagating on the extraordinary mode will mix in order to produce a 2ω wave propagating on the ordinary mode. The phase matching angle is calculated as the angle between a line, which contains both the origin and the intersection point between the sheets at ω and 2ω, and its projection on the $k_{1}k_{2}$ plane. The circle is parallel to the $k_{1}k_{2}$ plane and has $k_{3}$ passing through its center, making it a symmetry axis. Any plane containing $k_{3}$ sectioning the **k**-surface will result in a circle-ellipse configuration, with the point of intersection at the same angle of deviation with respect to the axis defined in the $k_{1}k_{2}$ plane by the sectioning plane. When changing the sign of the permittivity along one axis, say $\epsilon_{11}$, the associated **k**-surfaces are modified accordingly, as we have previously seen. As presented in Figure 8b, when increasing the value of $\epsilon_{11}$ and $\epsilon_{22}$, the intersection point between the two sheets at a given frequency shifts to a greater value as ω increases. Moreover, the geometric locus of the intersection between the sheets at ω and 2ω is defined by two curves: One curve is determined close to the intersection points of each individual **k**-surface, while the second is located further away, at greater permittivity values. In the case of negative dispersion, the **k**-surface at 2ω appears ‘displaced’ to lower values with respect to the one at ω: the intersection point of the two sheets at 2ω is closer to the origin than the one taken at ω. The curves describing the geometric locus of the intersections between the sheets at ω and 2ω retain their shape, but have their coordinate values decreased. In both cases, it is clear that there is no possible way of intersecting both curves with a single line coming from the origin, meaning that the obtained phase matching solutions are not degenerated. It is important to state that for this particular case, obtaining two phase matching curves instead of just one doubles the angular resolution of natural phase matching. Lastly, in the case of both ϵ values being negative, the single-sheet hyperboloids at ω and 2ω described in Figure 8c intersect each other, the intersection being a circle which is parallel to the $k_{1}k_{2}$ plane. The intersection circle also has $k_{3}$ as a symmetry axis, which implies that no matter the sectioning plane, the phase matching angle defined as before will have the same value, regardless of the direction of the cut.

**Figure 9:**
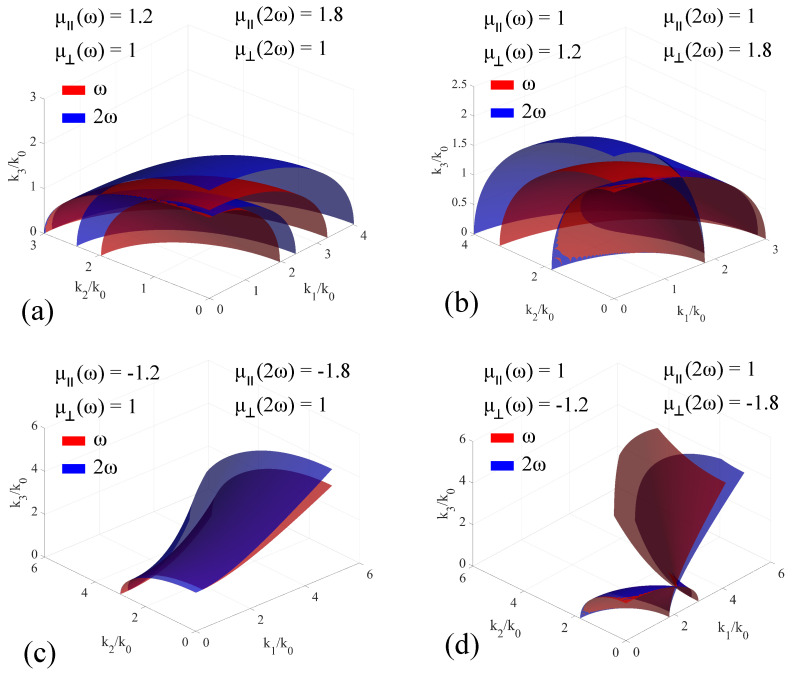
Extended **k**−surfaces for positive-valued permittivity artificial media exhibiting positive magnetic dispersion, for both positive− and negative−valued $\mu_{\|}$ and $\mu_{⊥}$. The permittivity values are $\epsilon_{11}=\epsilon_{22}=3.22$, $\epsilon_{33}=9$, and the medium is considered electrically non−dispersive, in order to highlight the modifications induced only by the magnetic response. The cases are: (**a**) $\mu_{\|}\left(\omega \right)=1.2$, $\mu_{\|}\left(2\omega \right)=1.8$, $\mu_{⊥}\left(\omega \right)=\mu_{⊥}\left(2\omega \right)=1$; (**b**) $\mu_{⊥}\left(\omega \right)=1.2$, $\mu_{⊥}\left(2\omega \right)=1.8$, $\mu_{\|}\left(\omega \right)=\mu_{\|}\left(2\omega \right)=1$; (**c**) $\mu_{\|}\left(\omega \right)=-1.2$, $\mu_{\|}\left(2\omega \right)=-1.8$, $\mu_{⊥}\left(\omega \right)=\mu_{⊥}\left(2\omega \right)=1$; (**d**) $\mu_{⊥}\left(\omega \right)=-1.2$, $\mu_{⊥}\left(2\omega \right)=-1.8$, $\mu_{\|}\left(\omega \right)=\mu_{\|}\left(2\omega \right)=1$.

**Figure 10:**
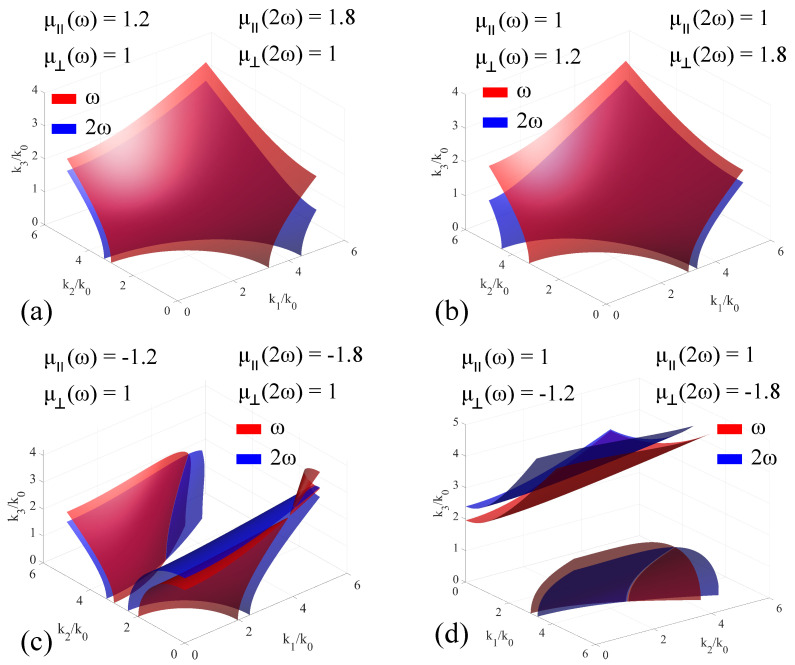
Extended **k**−surfaces for negative-valued permittivity artificial media exhibiting positive magnetic dispersion, for both positive− and negative−valued $\mu_{\|}$ and $\mu_{⊥}$. The permittivity values are $\epsilon_{11}=\epsilon_{22}=-3.22$, $\epsilon_{33}=9$, and the medium is considered electrically non−dispersive, in order to highlight the modifications induced only by the magnetic response. The cases are: (**a**) $\mu_{\|}\left(\omega \right)=1.2$, $\mu_{\|}\left(2\omega \right)=1.8$, $\mu_{⊥}\left(\omega \right)=\mu_{⊥}\left(2\omega \right)=1$; (**b**) $\mu_{⊥}\left(\omega \right)=1.2$, $\mu_{⊥}\left(2\omega \right)=1.8$, $\mu_{\|}\left(\omega \right)=\mu_{\|}\left(2\omega \right)=1$; (**c**) $\mu_{\|}\left(\omega \right)=-1.2$, $\mu_{\|}\left(2\omega \right)=-1.8$, $\mu_{⊥}\left(\omega \right)=\mu_{⊥}\left(2\omega \right)=1$; (**d**) $\mu_{⊥}\left(\omega \right)=-1.2$, $\mu_{⊥}\left(2\omega \right)=-1.8$, $\mu_{\|}\left(\omega \right)=\mu_{\|}\left(2\omega \right)=1$.

When considering magnetically-dispersive media, the **k**-surfaces change their shapes and relative position as a function of either $\mu_{\|}$ or $\mu_{⊥}$. In order to trace out the modification to the **k**-surface imparted by the positive-dispersion materials, the values of the electric permittivity were kept constant, with only the signs being cycled between positive and negative, for both the fundamental and the second harmonic. The artificial medium under consideration exhibits positive dispersion for both positive and negative-valued permeability components. For positive values of the electric permittivity, the obtained results are presented in Figure 9, while for negative values, the results are presented in Figure 10.

In the case of positive-valued ϵ and μ, we first assume that the magnetic response is exhibited only by $\mu_{\|}$, together with an associated positive dispersion. Apart from a modification of the direction of the optical axis for the two individual frequencies, which is to be expected, the two **k**-surfaces intersect each other in two points, corresponding to the optical axis of each **k**-surface. This case is presented in Figure 9a. When keeping $\mu_{\|}=1$ and attributing a response to the $\mu_{⊥}$ component, as well as a positive dispersion, the result mirrors the one above with respect to the $x_{3}$ axis. Just as before, phase matching is obtained at the optical axis of each surface. This case is depicted in Figure 9b. When changing the sign of the μ components, we obtain significant modification of the two surfaces: For negative-valued, positive-dispersion $\mu_{\|}$, the intersection of the single-sheet hyperbolas is a curve in the $k_{2}k_{3}$ plane, as presented in Figure 9c. For negative-valued, positive-dispersion $\mu_{⊥}$, the intersection of the two-sheet is created by displacing the 2ω surface with respect to the fundamental. The displacement increases with the dispersion coefficient, but is negligible for reasonably-small modification of $\mu_{⊥}$. From a phase-matching point of view, this behavior results in a relatively-low angular sensitivity across a broad spectrum, making such media appropriate for broadband mixing techniques. This configuration is presented in Figure 9d.

In the case of negative-valued ϵ, the magnetic property configurations are discussed as follows: For a positive-valued $\mu_{\|}$, the two hyperbolas are not intersecting each other in any point, meaning that collinear phase matching is not possible in this configuration. The associated **k**-surfaces are presented in Figure 10a. For a positive-valued $\mu_{⊥}$, the two **k**-surfaces are mirrored to the ones shown in configuration (a) with respect to the $x_{3}$ axis, and do not intersect each other. For negative-valued $\mu_{\|}$, the intersections of the two-sheet **k**-surfaces are two curves, one obtained from the intersection of the relatively-flat surfaces obtained at highter values of $k_{2}/k_{0}$, and the other obtained by the displacement of the inflection point belonging to each surface. As the frequency increases, this inflection point moves significantly towards higher-values of $k_{1}$, which indicates a higher angular sensitivity to broadband mixing. This configuration is presented in Figure 10c. For negative-valued $\mu_{⊥}$, the intersection of the two sheets is obtained only for the surfaces localized at low values of $k_{3}$, with the other sheets being displaced with respect to each other as $\mu_{⊥}$ increases. This configuration is presented in Figure 10d. Lastly regardless of the configuration used, introducing the chirality factor γ only introduces slight modifications in the behavior of the two sheets, and only if the chirality factor exhibits dispersion. As a result, the tuning curves that act as support for phase mixing in the collinear configuration are not modified in a significant way with the introduction of the chirality factor.

## 4. Conclusions

In this paper, we have theoretically characterized the set of possible behaviors exhibited by artificial media by means of extending the **k**-surface framework to negative-valued electromagnetic properties, as well as chiral media. Our framework finds a virtually-endless set of applications due to the possibility of creating artificial media with designer properties with individual sign and dispersion control of all the ϵ and μ components across each direction, as well as the possibility of artificially-introducing chirality in the desired medium. This remarkable control of every property was recently offered by metamaterials and metasurfaces. Apart from characterization of the properties at a given frequency, the study offers some insight into the nonlinear properties of the artificial media having the above electric and magnetic properties. Due to the fact that experimentally, the nonlinear efficiency is expressed by means of the collinearly-generated second harmonic generation, we have characterized the collinear phase matching condition for all the possible configurations, in the absence of a chirality factor. The importance of our work resides in the fact that our extended framework is able to provide easily-readable, direct information on the optical properties of the desired medium by providing the necessary estimates of $\hat{\epsilon }$, $\hat{\mu }$ and $\hat{n}$ components. Specifically, our framework is able to offer a graphic solution that is able to detect the presence of an optical axis under certain configurations or the presence of a phase matching tuning curve under specific conditions, given a set of input estimates. The framework is able to operate with positive and negative dispersion materials, and can account for the dispersion on each direction independently, offering a direct assessment of the optical properties under any possible combination of input components. The study can find applications in virtually any aspect of optical characterization of an artificial medium, either single-frequency or broadband, due to the fact that the framework presented here offers valuable predictions on the behavior of the artificial medium once it is designed.

## Data Availability

Not applicable.
